# Hypothalamic neuronal histamine mediates the thyrotropin-releasing hormone-induced suppression of food intake^1^

**DOI:** 10.1111/j.1471-4159.2007.04802.x

**Published:** 2007-11

**Authors:** Koro Gotoh, Koji Fukagawa, Tomiyo Fukagawa, Hitoshi Noguchi, Tetsuya Kakuma, Toshiie Sakata, Hironobu Yoshimatsu

**Affiliations:** Department of Internal Medicine 1, Faculty of Medicine, Oita University Yufu, Oita, Japan

**Keywords:** feeding behavior, histamine, hypothalamus, thyrotropin-releasing hormone

## Abstract

We examined the involvement of thyrotropin-releasing hormone (TRH) and TRH type 1 and 2 receptors (TRH-R1 and TRH-R2, respectively) in the regulation of hypothalamic neuronal histamine. Infusion of 100 nmol TRH into the rat third cerebroventricle (3vt) significantly decreased food intake (*p* < 0.05) compared to controls infused with phosphate- buffered saline. This TRH-induced suppression of food intake was attenuated partially in histamine-depleted rats pre-treated with α-fluoromethylhistidine (a specific suicide inhibitor of histidine decarboxylase) and in mice with targeted disruption of histamine H1 receptors. Infusion of TRH into the 3vt increased histamine turnover as assessed by pargyline-induced accumulation of tele-methylhistamine (t-MH, a major metabolite of neuronal histamine in the brain) in the tuberomammillary nucleus (TMN), the paraventricular nucleus, and the ventromedial hypothalamic nucleus in rats. In addition, TRH-induced decrease of food intake and increase of histamine turnover were in a dose-dependent manner. Microinfusion of TRH into the TMN increased t-MH content, histidine decarboxylase (HDC) activity and expression of HDC mRNA in the TMN. Immunohistochemical analysis revealed that TRH-R2, but not TRH-R1, was expressed within the cell bodies of histaminergic neurons in the TMN of rats. These results indicate that hypothalamic neuronal histamine mediates the TRH-induced suppression of feeding behavior.

Thyrotropin-releasing hormone (TRH) plays a fundamental role in the regulation of anterior pituitary TSH secretion (Yarbrough 1979). The endocrine function of TRH is supported by TRH type 1 receptors (TRH-R1) that are localized predominantly to hypothalamic regions. In addition, TRH-R1 expressed in other regions, such as the brain stem and spinal cord support a functional link between TRH and vegetative and autonomic functions. In contrast to the expression of TRH-R1, TRH type 2 receptor (TRH-R2) mRNA is distributed widely throughout the brain, including the thalamus, cerebral and cerebellar cortex, and the reticular formation. This distribution of TRH-R2 indicates that TRH is involved in higher cognitive functions as well as in regulating the arousal system, locomotor activity, and pain perception.

It was suggested recently that leptin plays an important role in the neuroendocrine regulation of the hypothalamic–pituitary–thyroid axis (Ahima *et al.* 1996; Legradi *et al.* 1997; Nillni and Sevarino 1999). TRH gene expression in the paraventricular nucleus (PVN) has been shown to be regulated by leptin (Flier and Maratos-Flier 1998; Mantzoros 1999; Nillni *et al.* 2000). In addition, neurons in the PVN that release TRH express both leptin and melanocortin-4 receptors, which suggests that TRH is an important part of the signaling systems that regulate feeding behavior. In line with this hypothesis, acute central administration TRH was reported to have anorexigenic effects (Vogel *et al.* 1979; Morley and Levine 1980; Breese *et al.* 1981).

Histamine is a neurotransmitter that regulates appetite, energy metabolism, body temperature, and the sleep–wake cycle (Doi *et al.* 1994; Sakata *et al.* 1997; Huang *et al.* 2001, 2006). We demonstrated previously that neuronal histamine suppressed food intake via H1 receptors within the PVN and ventromedial hypothalamus (VMH) (Fukagawa *et al.* 1989). Furthermore, hypothalamic neuronal histamine is one of the hypothalamic targets of leptin, as indicated by the observation that leptin accelerates the release of neuronal histamine within the hypothalamus (Yoshimatsu *et al.* 2001). Leptin-induced suppression of feeding is attenuated both in rats in which histamine has been depleted by α-fluoromethylhistidine (FMH), a suicide inhibitor of histamine synthesizing histidine decarboxylase (HDC) enzyme as well as in mice with targeted disruption of histamine H1 receptors (H1KO mice) (Yoshimatsu *et al.* 1998; Masaki *et al.* 2001). In contrast to the functional connection between histamine and leptin, little is known about how leptin activates neuronal histamine because the long form of the leptin receptor is not expressed by histaminergic neurons within the tuberomammillary nucleus (TMN) (Elmquist *et al.* 1998). As stated above, leptin elicits the expression of TRH in the PVN (Legradi *et al.* 1997). In addition, TRH immunoreactivity has been identified in some histamine-containing neurons within the TMN (Airaksinen *et al.* 1992). The aforementioned findings suggest that TRH might mediate leptin signaling to histaminergic neurons.

In the present study, we examined the neuroanatomical and functional relationship between TRH and neuronal histamine. We tested whether histamine depletion and targeted disruption of histamine H1 receptors attenuated the anorexic effects of TRH, if TRH increased histamine turnover within hypothalamic nuclei that are involved in energy homeostasis, and whether TRH receptors were expressed in histaminergic neurons within the TMN.

## Materials and methods

### Animals

We used adult (250–280 g) male Sprague–Dawley rats (Seac Yoshitomi, Fukuoka, Japan), and adult (25–30 g) male H1KO mice (C57BL/6 background). H1KO mice were generated by homogeneous recombination as described previously (Inoue *et al.* 1996). Animals were housed in a room illuminated daily from 7 am to 7 pm (12 h light–dark cycle) at a temperature of 21 ± 1°C and 55 ± 5% relative humidity. Animals were allowed free access to standard rat chow (CE-2; Clea Japan, Tokyo, Japan) and tap water. All rats were handled for 5 min on four successive days prior to each experiment to equilibrate the level of arousal (Sakata 1982). All studies were conducted in accordance with Oita University Guidelines for Animal Care, which are based on the National Institutes of Health Guide for the Care and Use of Laboratory Animals.

### Surgery

Under anesthesia (50 mg/kg sodium pentobarbital, i.p.), rats were placed in a stereotaxic apparatus (Narishige, Tokyo, Japan). A stainless steel guide cannula (23-gauge) was implanted chronically into the third cerebroventricle (3vt). This procedure was carried out at least 10 days before the start of infusions. A stainless steel wire stylet (30-gauge) was inserted into the guide cannula to prevent leakage of the CSF and obstruction of the cannula. A stainless steel cannula (23-gauge; 15 mm long) was inserted into the 3vt along the midline 6.0 mm anterior to the zero ear bar coordinate to a depth of 7.8 mm from the cortical surface according to the atlas of Paxinos and Watson (Paxinos and Watson 1986). Similarly, at least 1 week prior to the start of infusions, a cannula was implanted into the lateral cerebroventricle (lvt) of the mice 1.0 mm lateral from the midline and 0.22 mm posterior to the bregma at a depth of 2.0 mm from the cortical surface according to the atlas of Franklin and Paxinos (Franklin and Paxinos 1997). Details of the aforementioned surgical procedures have been described previously (Sakata *et al.* 1981).

### Drugs

TRH (10 μmol/mL), pargyline hydrochloride (0.33 mmol/kg), and FMH (50 mg/mL) (Sigma, St Louis, MO, USA) were dissolved in phosphate-buffered saline (PBS). Each solution was prepared freshly on the day of administration. The pH of each solution was adjusted to 6.5–7.5.

### Measurement of food intake

#### Change in food intake by different doses of TRH

Rats (*n* = 24) were divided into four groups (*n* = 6 per group). Food was removed 24 h before the experiment. On the day of the experiment, TRH (100 nmol/10 μL/10 min), TRH (10 nmol/10 μL/10 min), TRH (1 nmol/10 μL/10 min) or PBS were centrally infused. Food intake was measured for 1 h following the infusion of TRH or PBS.

#### Changes in food intake regulated by TRH after histamine depletion

Rats (*n* = 24) that were matched on the basis of body mass were allocated to one of four treatment groups (*n* = 6 rats per group). Food was removed 24 h before the experiment. On the day of the experiment, an equivalent volume of FMH (50 mg/kg) or PBS was administered (i.p.) 1 h before central infusion of TRH (100 nmol/10 μL/10 min) or PBS (10 μL/10 min). Food intake was measured for 1 h following the infusion of TRH or PBS.

#### Changes in food intake regulated by TRH in H1KO mice

Food was removed 24 h before the experiment. On the day of the experiment, TRH (10 nmol/μL) or PBS was infused via the lvt into H1KO (*n* = 12) or wild-type mice (*n* = 12) at a rate of 1.0 μL/min for 1 min. Food intake was measured for 1 h following the infusion of TRH or PBS.

#### Measurement of hypothalamic histamine and tele-methylhistamine content after central infusion of TRH or FMH

Rats were pre-treated with pargyline hydrochloride (0.33 mmol/kg, i.p.), an inhibitor of monoamine oxidase B which induced accumulation of tete-methylhistamine (t-MH) in the extraneural space as a major metabolite of released neuronal histamine 90 min before the central infusion of either TRH (100 nmol/10 μL/10 min), TRH (10 nmol/10 μL/10 min), TRH (1 nmol/10 μL/10 min), or PBS (10 μL/10 min). Different doses of TRH or PBS was infused centrally at a rate of 1 μL/min for 10 min to each group. Additional rats (*n* = 12) were divided into two groups (*n* = 6 per group). FMH (50 mg/kg) or PBS was intraperitoneally administered.

All rats were anesthetized with sodium pentobarbital (50 mg/kg, i.p.) before being exsanguinated by transcardiac perfusion with 100 mL saline that contained 200 units of heparin. After decapitation, the brain (including the encephalon) was removed and snap-frozen in liquid nitrogen. Brain regions that contained the lateral hypothalamus, PVN, VMH, and TMN were dissected using a frozen razor blade. The dissected tissue was submerged in 400 μL 0.5 M acetic acid before being homogenized. The homogenate was boiled for 10 min before a 50-μL sample was removed for a protein assay (Bio-Rad Laboratories, Hercules, CA, USA). Histamine content was measured in 50-μL samples using a histamine radioimmunoassay (RIA) kit (Eikenkagaku, Tokyo, Japan). The concentration of t-MH in each sample was measured in a 40-μL sample using a t-MH RIA kit (Pharmacia Upjohn, Tokyo, Japan), except FMH-infused group.

#### Measurement of HDC activity in the TMN and t-MH contents in the TMN, anterior hypothalamus and cortex after microinfusion of TRH into the TMN

Rats (*n* = 24) were used to measure HDC activity and brain t-MH levels following perfusion of TRH or PBS (6 rats per group) and additional 6 rats were used to verify the precise position of infusion. All rats were anesthetized with sodium pentobarbital (50 mg/kg, i.p.), placed in stereotaxic apparatus and unilaterally implanted with a stainless steel guide cannula (23-gauge; 15 mm long). TMN coordinates was −4.3 mm from bragma, lateral 1.5 mm to the midbrain, 8.0 mm below the cortical surface, according to the atlas of Paxinos and Watson (Paxinos and Watson 1986). Each guide cannula was placed with its tip 1 mm above its target site (unilateral side). A stainless wire stylet (29 gauge) was placed in the guide cannula until the testing day. A 16-mm-long infusion cannula (29 gauge) was unilaterally inserted into the guide cannula 30 min before testing so that the tip of the infusion cannula protruded 1 mm beyond the tip of the guide cannula. On the testing day, all rats were ascertained to have recovered to at least the pretreatment body weight. TRH (10 nmol/μL) or PBS was infused at the rate of 0.2 μL /min for 5 min (1 μL). Brain was removed and sections containing the TMN, anterior hypothalamus (AH) and cerebral cortex were dissected with a frozen razorblade. After homogenization, the concentration of tele-methylhistamine (t-MH) in each sample was measured using a t-MH RIA kit.

Rats treated with TRH (10 nmol/μL) or PBS (*n* = 6 per group) were used to measure the HDC activity in the TMN. HDC activity was evaluated by measuring the formation of histamine from L-histidine in the sample. After homogenization with 400 μL PBS and centrifugation at 1000 *g*, the reaction was started by addition of l-histidine (0.25 mmol/L) to the sample, incubated for 1 h. Histamine concentration was measured using a histamine RIA kit. Brains of the remaining rats (*n* = 6) were fixed by 4% paraformaldehyde and cut into 40-μm coronal sections through TMN.

#### Quantitative detection of rat HDC mRNA in the TMN by real-time reverse transcription polymerase chain reaction

On the testing day, TRH (1 nmol/μL), TRH (10 nmol/μL), TRH (50 nmol) or PBS was infused at the rate of 0.2 μL /min for 5 min (1 μL). Brain was removed and the section containing the TMN was dissected with a frozen razorblade. One-tube RT-PCR was performed using the Qiagen (Valencia, CA, USA) one-tube RT-PCR kit according to manufacturer’s recommendations. Primer sets included 5′-CCCAGTGAATACCATGAATAC-3′ (sense) and 5′-ATGTCCCCAAAGATGCTA-3′ (antisense) for rat HDC determination (designed based on GenBank accession number M29591). RT was performed at 50°C for 30 min. followed 40 cycles of PCR (95°C 30 s, 55°C 30 s, 72°C 30 s). Expression of each target gene was normalized to total RNA and samples run in triplicate. The standard curve samples used for RT-PCR were prepared by serial dilution of a RNA sample of known concentration to cover the range of 3000 to 3 ng. The log-linear portion of the standard curve was identified and regression coefficient *R*^2^ equaled 1.00. Threshold cycle (Ct) data were used to determine the amount of each target gene in the hypothalamus. ΔCt was defined as the average Ct value for PBS, TRH (1 nmol), TRH (10 nmol) or TRH (50 nmol) group, respectively. The percent change in expression, relative to the chow group, was defined as 2 –ΔΔCt × 100, where ΔΔCt equals the group ΔCt minus the ΔCt of PBS group. The SE of the ΔΔCt is considered the same as the SE of the ΔCt value because the calculation of ΔΔCt is subtraction of an arbitrary constant.

#### Immunohistochemistry

Rats were anesthetized with sodium pentobarbital (50 mg/kg, i.p.) before being exsanguinated by transcardiac perfusion with 50 mL saline that contained 50 units of heparin. This was followed by transcardiac perfusion with 300 mL 4% paraformaldehyde in ice-cold PBS. The brain was removed and divided into three segments (forebrain, diencephalon, and brain stem). The diencephalon was stored in PBS overnight. The tissue was then submerged in a 20% sucrose solution for 2 days, followed by submersion in a 30% sucrose solution for 3 days. Thereafter, the tissue was snap-frozen at −80°C before being sectioned at a thickness of 30 μm with a cryostat at −20°C. Sections were treated with 0.3% H_2_O_2_ in PBS for 30 min before being pre-soaked for 1 h in PBS that contained 0.3% Triton X-100 (Sigma), 2% bovine serum albumin, and 2% normal goat serum. The sections were incubated overnight at 4°C with polyclonal rabbit antiserum recognizing TRH-R1 or TRH-R2 (specificity for rat, 1 : 500; Santa Cruz Biotechnology Inc., CA, USA), followed by detection with biotin-conjugated goat anti-rabbit IgG and FITC-conjugated streptoavidin (ABC reagent; Vector Laboratories, Burlingame, CA, USA). Sections were then incubated overnight at 4°C with polyclonal rabbit antiserum recognizing HDC (specificity for rat, 1 : 2000; Chemicon Inc., Temecula, CA, USA) in a buffer that contained 0.3% Triton X-100 and 1% NGS. Sections were subsequently exposed to biotin-conjugated goat anti-rabbit IgG (ABC reagent; Vector Laboratories) and Rhodamine-conjugated streptoavidin (ABC reagent; Vector Laboratories). Negative control (single staining for HDC) was performed in each experiment, in which one of the primary antibodies (TRH-R1 or TRH-R2) was replaced with the normal serum, and further incubation with both secondary antibodies was performed as usual. Stained sections were examined with a confocal immunofluorescence microscope (Olympus, Tokyo, Japan). The area immunostained by antibodies were analyzed with a computed image analyzer (Lumina Vision, Mitsutani Corp. Kyoto, Japan). Rhodamine-stained area and FITC-stained area were visualized as red and green signals, respectively. The results are computer images of the same section that can be manipulated using reconstruction technique. Images were not adjusted or altered in any way, except for occasional adjustment of brightness.

### Statistics

Data are expressed as the mean ± SE. Differences were analyzed using anova followed by Fisher’s protected least-significant difference *post hoc* test. Significance was established at *p* < 0.05.

## Results

### Dose–response of TRH on food intake and contents of histamine and t-MH in the TMN

**Fig. 1:**
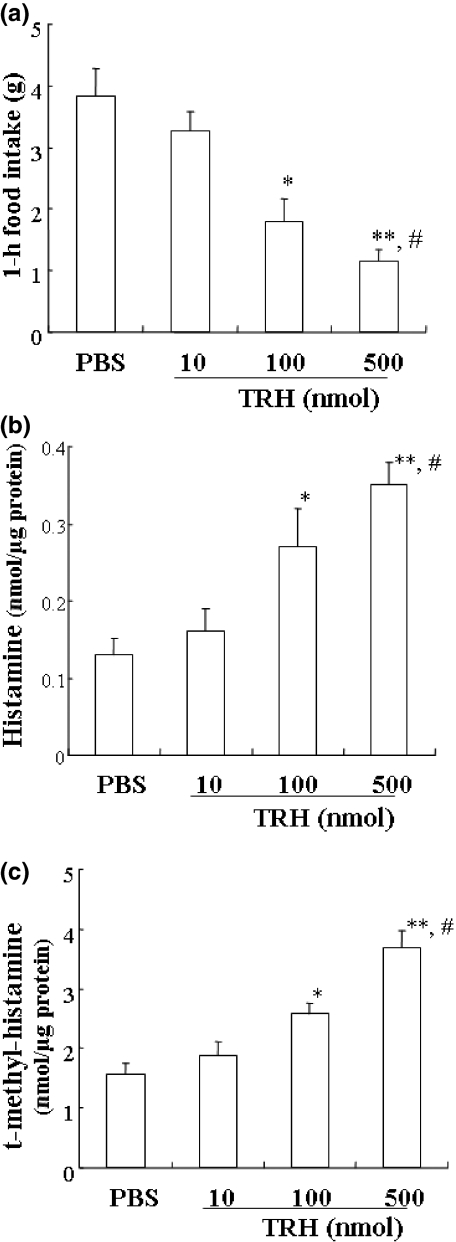
Dose effect of TRH on food intake and histamine contents in the TMN. (a) Food intake over 1 h in rats (*n* = 6 per group) after the central infusion of TRH at 10, 100 or 500 nmol or PBS. *p < 0.05 versus, **p < 0.01 versus PBS and TRH (10 nmol), #*p* ≤ 0.05 versus TRH (100 nmol). 3vt infusion of TRH at 100 and 500 nmol elevated the contents of histamine (b) and t-MH (c) in the TMN. (b) Histamine concentrations in the TMN of rats (*n* = 6 per group) treated by 3vt infusion with PBS or TRH (10, 100, or 500 nmol). (c) Concentrations of t-MH in the TMN of rats treated as described for (c). *p < 0.05 versus PBS and TRH (10 nmol), **p < 0.01 versus PBS and TRH (10 nmol), #*p* ≤ 0.05 versus TRH (100 nmol).

The central infusion of TRH at 100 and 500 nmol doses resulted in significant reduction of 1-hr food intake by about 50% (1.80 ± 0.36 vs. 3.85 ± 0.45 g and 1.17 ± 0.17 vs. 3.85 ± 0.45 g, respectively), compared to PBS (*p* < 0.05 and 0.01, respectively). Furthermore, TRH at the dose of 10–500 nmol decreased food intake in a dose-dependent manner. However, no significant suppression of 1-hr food intake was observed at 10 nmol of TRH, compared to PBS (3.27 ± 0.3 vs. 3.85 ± 0.45 g) (Fig. 1a).

We also examined the effect of different doses of TRH on histamine metabolism in the TMN of rats. As shown in Fig. 1b and c, the central infusion of TRH at 100 and 500 nmol doses increased histamine and t-MH contents in the TMN but not at 10 nmol of TRH (Fig. 1b and c). Thus, 100 nmol was the minimal effective dose of TRH to suppress feeding and increase histamine turnover in this experiment. Moreover, histamine and t-MH contents were also increased in a dose-dependent manner (Fig. 1b and c).

### Histamine content in the TMN after the FMH administration

**Fig. 2:**
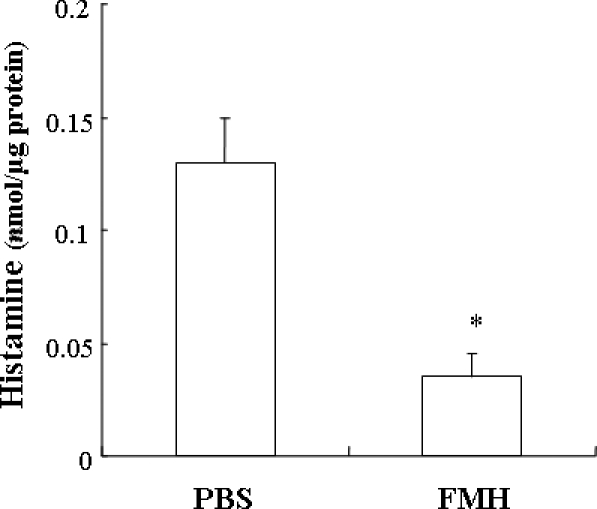
Effect of α-fluoromethylhistidine (FMH; 50 mg/kg, i.p.) on hypothalamic histamine content in the 2 h after the administration. The i.p. administration of FMH decreased histamine content in the TMN significantly. *p < 0.05 versus PBS.

Histamine content in the TMN after the administration of FMH (50 mg/kg) was significantly reduced by 73% in rats (*p* < 0.05) (Fig. 2). Therefore, FMH at 50 mg/kg dose is enough to deplete neuronal histamine in the TMN of the rat hypothalamus.

### Attenuation of the anorexic effects of TRH in histamine-depleted rats and H1KO mice

**Fig. 3:**
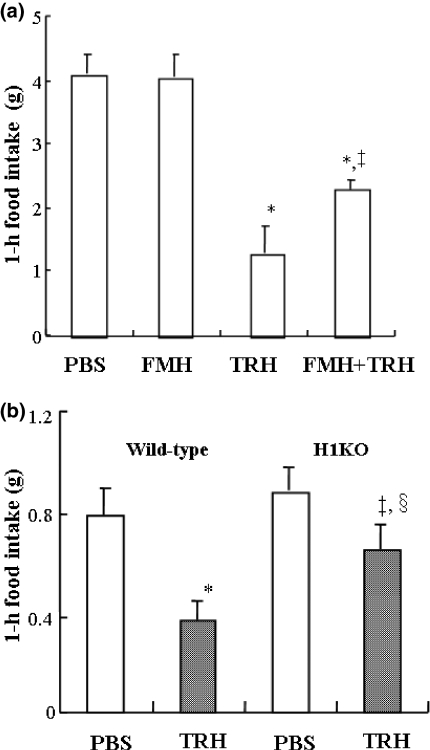
Anorexia induced by thyrotropin-releasing hormone (TRH) is attenuated in rats pre-treated with α-fluoromethylhistidine (FMH) and in mice with targeted disruption of the histamine H1 receptor (H1KO mice). (a) Food intake over 1 h in rats (*n* = 6 per group) treated with PBS (third cerebroventricle (3vt) infusion), FMH (50 mg/kg, i.p.), TRH (100 nmol; 3vt infusion), or both FMH and TRH. *p < 0.01 versus PBS-treated controls and FMH-treated controls. ^‡^*p* < 0.05 versus TRH-treated rats. (b) Food intake over 1 h in H1KO mice (*n* = 6 per group) treated with PBS (lateral ventricle infusion) or TRH (100 nmol; lvt infusion). *p < 0.05 versus PBS-treated controls (wild-type mice). ^‡^*p* < 0.05 versus PBS-treated controls (H1KO mice). ^§^*p* < 0.05 versus TRH-treated wild-type mice.

Food intake in rats whose hypothalamic histamine content was increased by bolus administration of TRH (100 nmol) into the 3vt (1.34 ± 0.43 g) was lower (*p* < 0.01) than that in control (PBS-treated) rats (4.14 ± 0.29 g; *n* = 6 per group). This anorexic effect of TRH was significantly attenuated by pretreatment with FMH, which was associated with a significantly smaller reduction in food intake (2.34 ± 0.16 g; *p* < 0.05) compared to rats treated with TRH alone. Administration of FMH alone did not affect food intake (Fig. 3a). Similarly, TRH caused a 50% reduction in food intake in control (wild-type) mice (*p* < 0.01), whereas the same treatment of histamine-depleted H1KO mice caused a significantly smaller (30%; *p* < 0.05) reduction in food intake (Fig. 3b).

### Effects of TRH on hypothalamic histamine and t-MH content

**Fig. 4:**
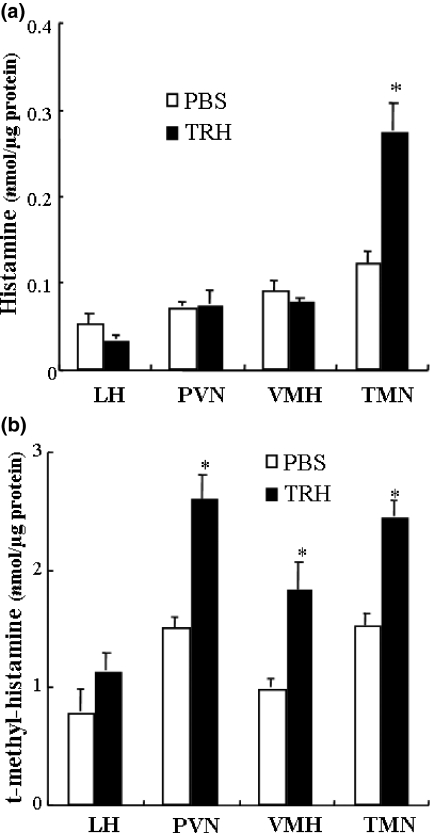
Effects of TRH on hypothalamic histamine and telemethylhistamine (t-MH) content. 3vt infusion of TRH elevated the concentration of histamine within the tuberomammillary nucleus (TMN) (a) and increased the concentration of t-MH in the TMN, paraventricular nucleus, and ventromedial hypothalamus (b). (a) Histamine concentrations in the hypothalamic nuclei of rats (*n* = 6 per group) treated by 3vt infusion with PBS or TRH (100 nmol). (b) Concentrations of t-MH in the hypothalamic nuclei of rats treated as described for (a). *p < 0.05 versus PBS-treated controls.

Central infusion of TRH increased the concentration of histamine in the TMN (*p* < 0.01 versus control; Fig. 4a) and increased the pargyline-induced accumulation of t-MH in the TMN, VMH, and PVN in rats (*p* < 0.05 versus control; Fig. 4b).

### Effect of TRH administration into the TMN on t-MH contents, HDC activity and HDC mRNA expression in the TMN

**Fig. 5:**
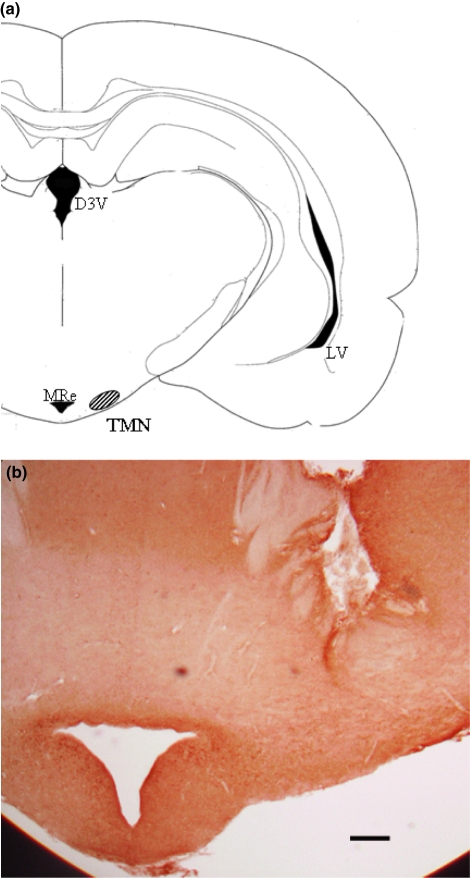
Schema indicating the area of TMN in the hypothalamus and histological identification of cannula tips in a representative infusion site stained by histamine neurons. TMN, tuberomammillary nucleus; D3 V, dorsal third ventricle; LV, lateral ventricle; MRe, mammillary recess of third ventricle. Tip of cannula was just above the TMN. Scale bar: 200 μm.

**Fig. 6:**
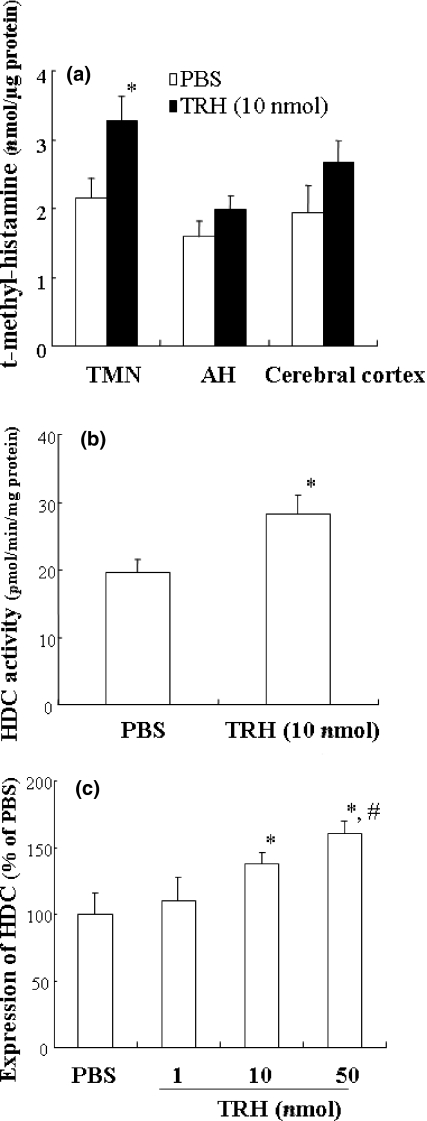
Effects of TRH injection into the TMN of the hypothalamus on tele-methylhistamine (t-MH) contents, HDC activity and HDC mRNA expression in the TMN. (a) Microinfusion of TRH (10 nmol) into the TMN of the hypothalamus elevated the t-MH contents in the TMN, but not in the AH and cerebral cortex. *p < 0.05 versus PBS-treated controls. (b) Microinfusion of TRH (10 nmol) into the TMN significantly increased HDC activity in the TMN. *p < 0.05 versus PBS infusion. (c) Expression of HDC in the TMN of TRH (1, 10, or 50 nmol) and PBS groups with real-time RT-PCR. *p ≤ 0.05 versus PBS and TRH (1 nmol) infusion, #*p* ≤ 0.05 versus TRH (10 nmol) infusion.

Placement of the cannula was compared to the atlas of Paxinos and Watson (Fig. 5a) (Paxinos and Watson 1986). Fig. 5b shows a typical TRH-infused site in rats. It was verified that the tip of guide cannula was just above the TMN. No chronically implanted guide cannula was found to touch or damage the tissue of a target locus. The insertion of the thin infusion cannula did not cause excessive tissue destruction. Microinfusion of TRH into the TMN increased the t-MH content in TMN of the hypothalamus, but not in the AH and cerebral cortex, compared with PBS infusion (*p* < 0.05; Fig. 6a). Correspondingly, the activity of HDC was also increased by administration of TRH compared with PBS infusion (*p* < 0.05; Fig. 6b). Moreover, HDC expression was increased by microinfusion of TRH at 10 and 50 nmol into the TMN but not at 1 nmol dose of TRH. Moreover, TRH at the dose of 1–50 nmol increased HDC expression in a dose-dependent manner (Fig. 6c).

### Immunohistochemical detection TRH-R1 and TRH-R2 in histaminergic neurons

**Fig. 7:**
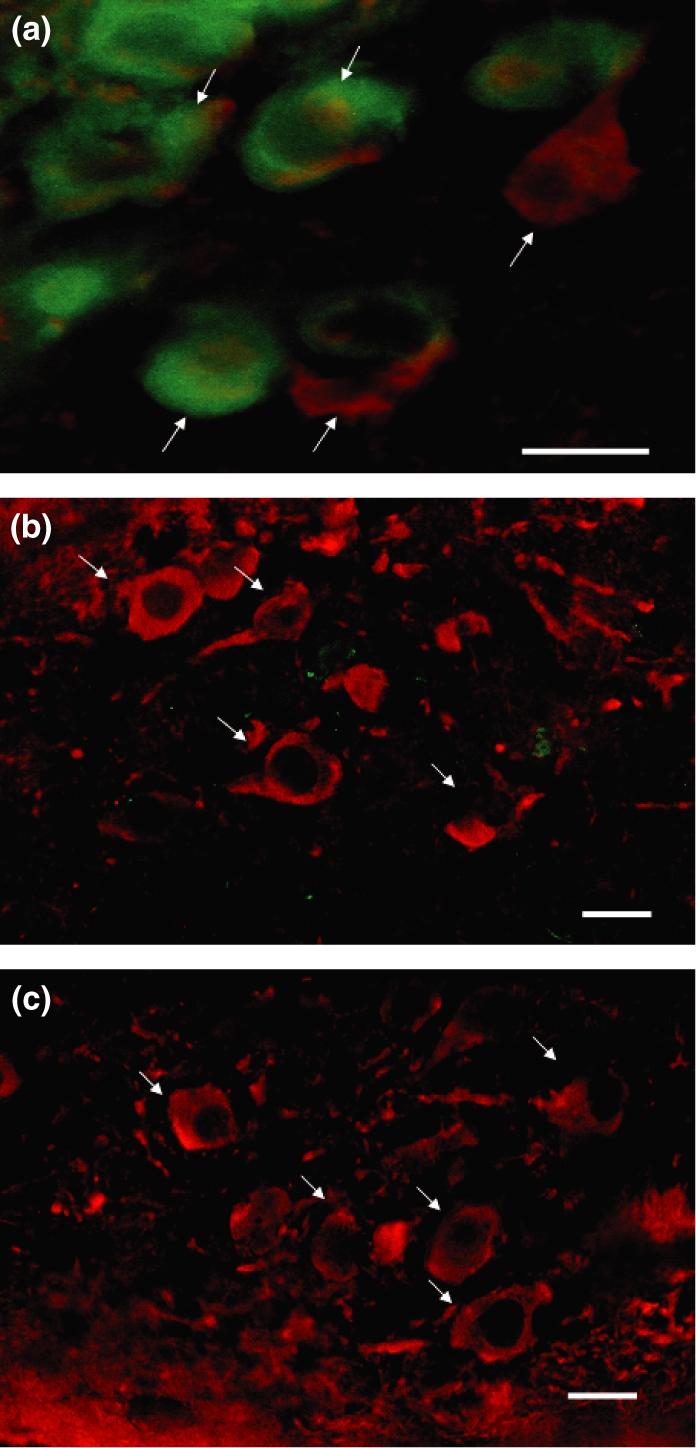
Immunohistochemical detection of TRH receptors in histaminergic neurons within the TMN. (a) Histaminergic neurons (red, white arrows) expressed TRH type 2 receptors (green). (b) Histaminergic neurons (red, white arrows) did not express TRH type 1 receptors (green). (c) Substitution of TRH-R1 or TRH-R2 antibodies with normal serum and incubation with secondary antibody as for double staining results in no detectable signal. Scale bar: 10 μm.

Specific antibodies revealed that TRH-R2 (green) was expressed within histaminergic cell bodies in the TMN of rats (red, white arrows) (Fig. 7a), whereas there was no expression of TRH-R1 (green) in histaminergic cell bodies (red, white arrows) (Fig. 7b). The specificity of the labeling was controlled by omitting the TRH-R1 or TRH-R2 antiserum. (Fig. 7c).

## Discussion

The present study demonstrated that the central infusion of TRH at 100 nmol not but 10 nmol induced suppression of food intake. This finding is consistent with the previous study showing that the central infusion of TRH (25, 50, 100 nmol) suppressed starvation-induced feeding in a dose related manner (Suzuki *et al.* 1982). Moreover, the depletion of neuronal histamine by pre-treatment with FMH attenuated TRH-induced suppression of food intake. These results suggest that endogenous neuronal histamine may mediate the suppression of food intake by TRH. We showed previously that hypothalamic neuronal histamine suppressed food intake via H1 receptors within the VMH and PVN, both of which are nuclei that are central to satietiation (Oohara *et al.* 1994). Our finding in the present study that the anorexigenic effect of TRH was also attenuated in H1KO mice is further evidence that histamine and H1 receptors mediate TRH signaling to suppress food intake.

The involvement of TRH–histamine signaling in suppressing food intake was also demonstrated by our study that hypothalamic histamine and t-MH concentrations exhibited region-specific increased in response to the administration of TRH and this effect of TRH was dose-dependent in the range of 10 to 500 nmol. Specifically, the increase in histamine concentrations was restricted to the TMN and was not detected in any other hypothalamic nuclei. After pretreatment with pargyline hydrochloride, infusion of TRH into the 3vt increased the concentration of t-MH not only in the TMN but also in the PVN and VMH, which indicated that TRH induced an increase in histamine turnover within these nuclei.

It might be suggested TRH infusion did not affect concentration of hypothalamic histamine in the hypothalamic nucleus except the TMN because histamine levels measured in present study included released histamine in the extraneuronal space as well as intracellular histamine in the nerve terminal and histamine is rapidly converted to its metabolite, t-MH, in the brain (Oohara *et al.* 1994).

Moreover, microinfusion of TRH into the TMN increased HDC activity and t-MH content in the TMN. The transmethylation of histamine into t-MH, a major metabolite of histamine, and its subsequent deamination, is the major metabolic pathway of histamine in the brain. Therefore, it is much more informative to analyze histamine release by measuring its metabolite than to rely on a measurement of the brain histamine level itself. Pre-treatment with pargyline, an inhibitor of monoamine oxidase B, is useful for this assessment, as it induces the accumulation of t-MH in the extraneuronal space (Oishi *et al.* 1987; Oohara *et al.* 1994, 1994).

The TMN contains the cell bodies of histaminergic neurons, whereas the PVN and VMH are major projection sites of histamine (Sakata and Yoshimatsu 1997). Based on this anatomical scheme, we conclude that TRH increased histamine turnover not only in cell bodies within the TMN but also at the nerve terminals within the PVN and VMH. The possibility that TRH directly modulated histamine release at nerve terminals in these sites cannot be excluded; however, it is likely that TRH activates histaminergic neurons within the TMN, and thereafter induces histamine release from the nerve terminals at projection sites because TRH receptors are expressed on the cell bodies of histaminergic neurons within the TMN.

TRH-R1 and TRH-R2 exhibit distinct expression patterns within the brain. In general, TRH-R1 is localized to the hypothalamus and brain stem, which supports a link between TRH-R1 and vegetative and autonomic functions. In contrast, TRH-R2 mRNA is distributed widely throughout the brain, including the cerebral cortex and reticular formation, which indicates that TRH-R2 may be involved in regulating cognition and the arousal system. In the present study, we identified TRH-R2, but not TRH-R1, within the cell bodies of histaminergic neurons within the TMN. TRH-containing neurons project to the TMN (Airaksinen *et al.* 1992), and it is well established that hypothalamic neuronal histamine regulates the sleep–wake cycle via connections to the ventrolateral preoptic area (the “sleep center”) and orexinergic neurons within the lateral hypothalamic area (Mignot *et al.* 2002; Huang *et al.* 2007). Therefore, the TRH–TRH-R2–histamine pathway may constitute a neuronal network that regulates arousal. In addition, our findings suggest that this pathway also contributes to the effects of TRH on feeding behavior.

Present study suggests that both orexin and TRH may be able to stimulate histaminergic system. Orexin increases food intake whereas TRH decreases it. Previous study reported that the central administration of orexin-A caused a significant increase in locomotor activity, but not food intake (Ishizuka *et al.* 2006). From these findings, it may be concluded that the histaminergic system participates in arousal state rather than feeding regulation by orexinergic neurons. On the other hand, histaminergic system mediates the TRH-induced suppression of feeding behavior partially. The mechanism involved in the anorectic effect of TRH except histaminergic system is uncertain, but it is possibility that TRH might cause anorectic effect by activating TRH receptors localized in the PVN and VMH, regions known to be involved in control of food intake and the site of the so-called satiety center, directly.

Leptin acts on the brain to inhibit food intake (Campfield *et al.* 1995). 3vt infusion of leptin increases histamine turnover rate (Yoshimatsu *et al.* 2001). Leptin-induced suppression of feeding was reported to be attenuated both in rats whose histamine was depleted by FMH, a suicide inhibitor of histamine synthesizing HDC enzyme, and in H1KO mice (Yoshimatsu *et al.* 1998; Masaki *et al.* 2001). Despite these findings, how leptin activates histamine-containing neurons within the hypothalamus is not known. Leptin receptor mRNA is expressed within the PVN (which is where the cell bodies of neurons that release TRH are located) but not in the TMN (which is the origin of histaminergic neurons) (Flier and Maratos-Flier 1998; Mantzoros 1999; Nillni *et al.* 2000). Recently, we reported that neurons that contain glucagon-like peptide 1 (GLP-1) and corticotropin-releasing hormone may mediate leptin signaling to histaminergic neurons within the TMN (Gotoh *et al.* 2005). The results of the present study suggest that there is an additional pathway that transmits leptin signals to the histaminergic system. Because leptin increases TRH expression in the PVN (Flier and Maratos-Flier 1998; Mantzoros 1999; Nillni *et al.* 2000), and in light of our finding a functional and neuroanatomical link between TRH and neuronal histamine, it is likely that leptin may activate histaminergic neurons indirectly via neurons that release TRH.

## Abbreviations used

TRH: thyrotropin-releasing hormone
3vt: third cerebroventricle
TMN: tuberomammillary nucleus
HDC: histidine decarboxylase
VMH: ventromedial hypothalamus
PVN: paraventricular nucleus
FMH: α-fluoromethylhistidine
PBS: phosphate-buffered saline
RIA: radioimmunoassay
